# Three-dimensional gait analysis of lower extremity gait parameters in Japanese children aged 6 to 12 years

**DOI:** 10.1038/s41598-022-11906-1

**Published:** 2022-05-12

**Authors:** Tadashi Ito, Koji Noritake, Yuji Ito, Hidehito Tomita, Jun Mizusawa, Hiroshi Sugiura, Naomichi Matsunaga, Nobuhiko Ochi, Hideshi Sugiura

**Affiliations:** 1 Three-Dimensional Motion Analysis Room, Aichi Prefectural Mikawa Aoitori Medical and Rehabilitation Center for Developmental Disabilities, 9-3 Koyaba Kouryuji Cho, Okazaki, 444-0002 Japan; 2 Department of Integrated Health Sciences, Graduate School of Medicine, Nagoya University, Nagoya, Japan; 3 Department of Orthopedic Surgery, Aichi Prefectural Mikawa Aoitori Medical and Rehabilitation Center for Developmental Disabilities, Okazaki, Japan; 4 Department of Pediatrics, Nagoya University Graduate School of Medicine, Nagoya, Japan; 5 Department of Pediatrics, Aichi Prefectural Mikawa Aoitori Medical and Rehabilitation Center for Developmental Disabilities, Okazaki, Japan; 6 Graduate School of Health Sciences, Toyohashi SOZO University, Toyohashi, Japan; 7 Department of Rehabilitation, Aichi Prefectural Mikawa Aoitori Medical and Rehabilitation Center for Developmental Disabilities, Okazaki, Japan

**Keywords:** Health care, Medical research, Neurology

## Abstract

We aimed to develop gait standards for gait parameters in school-aged Japanese children and assess age-related differences in gait patterns and parameters. Children aged 6–12 years (n = 424) were recruited from two elementary schools. An instrumented three-dimensional gait analysis system was used to record each child's gait kinematics, kinetics, and spatiotemporal parameters. Participants were subdivided into three age groups (Group A, 6–8 years; Group B, 9–10 years; and Group C, 11–12 years). LMS Chartmaker, version 2.54, was used to create a developmental chart for the gait pattern. The non-normalized step and stride lengths were significantly longer, and the cadence was lower in older children; however, the opposite outcome occurred when analyzing normalized data. Ankle moment differed significantly by age, and the maximum ankle moment was higher in older children than that in younger children. Furthermore, the hip and knee flexion angles during gait and the normalized spatiotemporal parameters of Japanese children aged 6–12 years differed by age and from those of children from other countries. The centile chart of the gait pattern is a useful tool for clinicians to assess developmental changes in the gait pattern and detect gait abnormalities in children.

## Introduction

Gait forms a complex but unconscious motor pattern that is an integral part of life, allowing individuals to function within the human environment and participate in daily activities. Gait ability is a critical element in measuring the quality of life and reflects a healthy individual’s status^1,2^. Therefore, it is important to characterize the kinematics and kinetics of gait in children according to age to assess normal gait parameters. Furthermore, the maturation of gait kinematics and kinetics in typically developing children is a key concern, so normative values are needed to assess normal development. Thus, it is important to build a reference database of age-related gait changes in children.

Gait analysis is a component of physical function assessment that can be used to screen for gait impairments and abnormalities^3–6^. Additionally, gait analysis is an important clinical tool for evaluating normal and pathological gait patterns, and it can be used to facilitate treatment-related decision-making and assess intervention outcomes^7–11^. Several studies have generated reference data on gait parameters in children^12–15^. However, data on age-related gait parameters in children with typical development remain scarce^16^.

Research on gait using three-dimensional (3D) motion analysis has revealed that children aged 7–11 years exhibit adult-like gait patterns. These findings are supported by research on joint kinematics^14,17,18^. Furthermore, most studies on kinematics and kinetics suggest that parameters begin to stabilize at approximately 5 or 7 years of age^12–14,19,20^. A study on gait parameters in children aged 4–13 years revealed that gait parameters are a reliable measure of gait maturation^19^. Creating developmental centile charts of gait parameters enables deviations from a typical developmental trajectory to be identified^21^. Thus, developmental centile charts of gait parameters could be used to assess childhood development.

To our knowledge, no studies have been conducted to assess the kinematics and kinetics of 3D gait analysis in Japanese children with typical development. Additionally, there are no reference data on the gait parameters of Japanese children with typical development. Gait patterns and parameters in humans are reported to vary by country, race, and age^8,12,13,17,22,23^. Hence, a Japanese reference database of normative kinematic and kinetic values is needed to assess how gait is affected by gait abnormalities in Japanese children and to allow comparisons with children in other countries. Furthermore, an assessment of the age at which gait kinematics and kinetics stabilize requires observation of a large population of children. The primary objective of this study was to generate a reference database on gait kinematics, kinetics, and spatiotemporal parameters in Japanese children and create developmental centile charts of gait patterns for clinicians. Furthermore, the study aimed to assess age-related differences in the aforementioned gait parameters between three age groups^8,12,13,17,22,23^. To date, studies on changes in anthropometric measurements have reported that children’s step and stride lengths, speed, and cadence continue to evolve until the child is fully grown^8,17,24^. In Japan, elementary school comprises six grades, with the first two grades being attended by 6–8 year-olds, the middle two grades being attended by those 8–10 years of age, and the last two grades being attended by 10–12-year-olds. Based on this fact, as well as previous studies that considered growth-related changes^25^ and the gait analysis research conducted by Smith et al.,^8^ the children were divided into the following three age groups: 6–8 (Group A), 9–10 (Group B), and 11–12 years (Group C).

## Results

Between January 2018 and March 2020, 437 children participated in the study, 432 of whom met the basic inclusion criteria. In total, 424 children aged 6–12 years were included in the analysis after excluding eight children meeting the exclusion criteria. Table 1 presents the participant characteristics. Values for all variables except for sex were significantly higher among older children; there was no significant between-group difference with respect to sex.

**Table 1:** Demographic characteristics of the participants (N = 424).

Variables	Group A (n = 141)	Group B (n = 154)	Group C (n = 129)	*P* value	Effect size (η^2^) or Cramer V
Age (years)	7.0 (6–8)	9.0 (9–10)	12.0 (11–12)	A and B: < 0.0001	0.91
				A and C: < 0.0001	
				B and C: < 0.0001	
Sex, n (%)				0.819	0.03
Boys	69 (48.9)	73 (47.4)	66 (51.2)		
Girls	72 (51.1)	81 (52.6)	63 (48.8)		
Height (cm)	123.5 (106.5–136.0)	134.6 (119.6–148.7)	147.8 (131.7–164.2)	A and B: < 0.0001	0.73
				A and C: < 0.0001	
				B and C: < 0.0001	
Weight (kg)	22.3 (16.1–38.0)	29.1 (20.5–45.1)	37.0 (24.8–74.4)	A and B: < 0.0001	0.59
				A and C: < 0.0001	
				B and C: < 0.0001	
Body mass index (kg/m^2^)	14.83 (12.98–22.01)	15.80 (12.32–26.62)	16.88 (13.35–29.60)	A and B: < 0.0001	0.18
				A and C: < 0.0001	
				B and C: < 0.0001	
Leg length (m)	0.59 (0.50–0.70)	0.66 (0.57–0.76)	0.75 (0.64–0.86)	A and B: < 0.0001	0.71
				A and C: < 0.0001	
				B and C: < 0.0001	

Group A (6–8 years), Group B (9–10 years), Group C (11–12 years).

Differences in age, height, weight, body mass index, and leg length, but not sex, between the three groups were analyzed using the Kruskal–Wallis test. Variables with significant differences were subsequently compared using multiple comparison analyses with Bonferroni correction. Data are presented as median values (ranges). The *P* value for sex was derived using the Chi-square test. *P* values < 0.0001 were considered statistically significant.

Tables 2 and 3 presents the non-normalized and normalized values of the spatiotemporal parameters by age group, and the *P* values for between-group comparisons. In the analysis of the non-normalized data, there was a significant increase in step and stride lengths with age. There were also significant differences in cadence, step time, stride time, single support, and double support between Groups A and C and between Groups B and C. The difference in gait speed between Groups A and B and Groups A and C increased with age. Group C had a higher Gait Deviation Index (GDI) value than Group A. The GDI is commonly used to quantitatively detect gait kinematics; for example every 10-point decrease from 100 points represents a decline of one standard deviation from the normal value. In the analysis of the normalized parameters, there were significant differences in step and stride length between Groups B and C (Table 3). Furthermore, there were significant differences in cadence, step time, stride time, and single support between Groups A and C. However, there were no significant differences in gait speed or double support between the groups.

**Table 2:** Non-normalized spatiotemporal parameters and Gait Deviation Index for each age group (N = 424).

Variables	Group A (n = 141)	Group B (n = 154)	Group C (n = 129)	*P* value	Effect size (η^2^)
Cadence (steps/min)	130.36 (93.50–164.82)	127.20 (105.50–161.06)	121.76 (91.06–151.19)	A and B: 0.162	0.13
				A and C: < 0.0001	
				B and C: < 0.0001	
Gait speed (m/s)	1.09 (0.65–1.57)	1.21 (0.75–1.75)	1.22 (0.84–1.77)	A and B: < 0.0001	0.12
				A and C: < 0.0001	
				B and C: < 1.000	
Step length (m)	0.50 (0.38–0.67)	0.57 (0.42–0.69)	0.60 (0.45–0.80)	A and B: < 0.0001	0.33
				A and C: < 0.0001	
				B and C: < 0.0001	
Stride length (m)	1.01 (0.77–1.32)	1.14 (0.84–1.37)	1.20 (0.89–1.60)	A and B: < 0.0001	0.33
				A and C: < 0.0001	
				B and C: < 0.0001	
Step time (s)	0.46 (0.35–0.64)	0.47 (0.37–0.57)	0.49 (0.40–0.66)	A and B: 0.098	0.13
				A and C: < 0.0001	
				B and C: < 0.0001	
Stride time (s)	0.92 (0.73–1.29)	0.94 (0.75–1.14)	0.99 (0.80–1.32)	A and B: 0.178	0.13
				A and C: < 0.0001	
				B and C: < 0.0001	
Single support (s)	0.39 (0.23–0.50)	0.40 (0.19–0.46)	0.41 (0.34–0.53)	A and B: 0.053	0.12
				A and C: < 0.0001	
				B and C: < 0.0001	
Double support (s)	0.14 (0.07–0.30)	0.15 (0.08–0.23)	0.16 (0.07–0.27)	A and B: 1.000	0.06
				A and C: < 0.0001	
				B and C: < 0.0001	
Gait Deviation Index (points)	93.37 (6.92)	93.82 (7.73)	96.82 (7.35)	A and B: 1.000	0.04
				A and C: < 0.0001	
				B and C: 0.002	

Group A (6–8 years), Group B (9–10 years), Group C (11–12 years).

Differences between the three groups were analyzed using the Kruskal–Wallis test or one-way analysis of variance. Variables with significant differences were subsequently compared using multiple comparison analyses with Bonferroni correction. Data are presented as means (standard deviation) or median values (range). *P* values < 0.0001 were considered statistically significant.

**Table 3:** Normalized spatiotemporal parameters for each age group (N = 424).

Variables	Group A (n = 141)	Group B (n = 154)	Group C (n = 129)	*P* value	Effect size (η^2^)
Cadence (steps/min)	32.11 (23.88–40.27)	33.21 (27.14–42.55)	33.36 (26.65–41.24)	A and B: 0.002	0.04
				A and C: < 0.0001	
				B and C: 1.000	
Gait speed (m/s)	0.45 (0.26–0.66)	0.48 (0.30–0.66)	0.45 (0.31–0.64)	0.003	0.03
Step length (m)	0.85 (0.09)	0.86 (0.08)	0.81 (0.08)	A and B: 1.000	0.05
				A and C: 0.002	
				B and C: < 0.0001	
Stride length (m)	1.70 (0.16)	1.71 (0.16)	1.62 (0.16)	A and B: 1.000	0.05
				A and C: < 0.001	
				B and C: < 0.0001	
Step time (s)	1.87 (1.49–2.50)	1.82 (1.43–2.21)	1.80 (1.46–2.26)	A and B: 0.006	0.04
				A and C: < 0.0001	
				B and C: 1.000	
Stride time (s)	3.74 (3.00–5.04)	3.62 (2.82–4.43)	3.60 (2.92–4.51)	A and B: 0.002	0.05
				A and C: < 0.0001	
				B and C: 1.000	
Single support (s)	1.58 (1.01–1.96)	1.53 (0.71–1.79)	1.52 (1.25–1.88)	A and B: < 0.001	0.07
				A and C: < 0.0001	
				B and C: 0.182	
Double support (s)	0.58 (0.29–1.16)	0.57 (0.31–0.84)	0.59 (0.21–0.91)	0.134	0.01

Group A (6–8 years), Group B (9–10 years), Group C (11–12 years).

Differences between the three groups were analyzed using the Kruskal–Wallis test or one-way analysis of variance. Variables with significant differences were subsequently compared using multiple comparison analyses with Bonferroni correction. Data are presented as means (standard deviation) or median values (range). *P* values < 0.0001 were considered statistically significant.

Tables 4, 5, and 6 present the values for the range of motion and the maximum and minimum values of the lower limb kinematics and kinetics during the gait cycle. There was a statistically significant difference in the maximum hip flexion values of the stance phase between Groups B and C; Group B had more relative flexion at the hip joint in the stance phase than Group C. The difference in the maximum knee flexion values of the swing phase between Groups A and C and Groups B and C decreased with age; Groups A and B had more relative flexion at the knee joint in the swing phase than Group C, although the magnitude of the change decreased with age.

**Table 4:** The minimum and maximum values for the gait kinematics of the pelvis and hip angle for the three age groups (N = 424).

Variables	Group A (n = 141)	Group B (n = 154)	Group C (n = 129)	*P* value	Effect size (η^2^)
Pelvis tilt anterior/posterior stance phase angle (degrees)	Minimum 12.68 (4.69) maximum 15.36 (4.52)	Minimum 13.46 (4.35) maximum 16.24 (4.45)	Minimum 11.64 (3.98) maximum 14.44 (3.85)	Minimum 0.002 maximum 0.002	Minimum 0.03 maximum 0.03
Pelvis obliquity upward/downward stance phase angle (degrees)	Minimum − 4.49 (− 8.98 to − 1.22) maximum 5.22 (2.53–8.95)	Minimum − 4.68 (− 9.12 to − 1.59) maximum 5.66 (1.21–9.51)	Minimum − 4.36 (− 9.45 to − 2.24) maximum 5.20 (2.89–9.46)	Minimum 0.392 maximum 0.224	Minimum 0.004 maximum 0.01
Pelvis Rotation internal/external rotation angle (degrees)	Minimum − 6.43 (− 17.72 to − 1.13) maximum 7.63 (1.65–14.99)	Minimum − 6.59 (− 20.10 to − 1.63) maximum 7.65 (2.63–19.84)	Minimum − 6.23 (− 13.57 to − 1.17) maximum 7.09 (2.10–16.84)	Minimum 0.119 maximum 0.021	Minimum 0.01 maximum 0.02
Hip flexion/extension stance phase angle (degrees)	Minimum − 6.11 (5.89) maximum 37.48 (18.09–55.00)	Minimum − 5.15 (4.90) maximum 38.47 (25.41–54.60)	Minimum − 6.11 (5.06) maximum 35.87 (22.92–50.67)	Minimum 0.202 maximum A and B: 0.214 A and C: 0.056 B and C: < 0.0001	Minimum 0.01 maximum 0.04
Hip adduction/abduction stance phase angle (degrees)	Minimum − 5.61 (− 14.71–1.11) maximum 6.13 (0.08– 10.97)	Minimum − 6.12 (− 14.09 to − 0.87) maximum 5.72 (0.14– 10.44)	Minimum − 6.36 (− 15.34–1.31) maximum 5.41 (− 0.83– 9.94)	Minimum 0.248 maximum 0.006	Minimum 0.01 maximum 0.02
Hip internal/external rotation stance phase angle (degrees)	Minimum − 10.74 (− 22.39–0.74) maximum 4.83 (− 8.60–15.31)	Minimum − 11.29 (− 28.19 to − 1.00) maximum 2.54 (− 9.83–15.56)	Minimum − 9.81 (− 28.7 to − 2.96) maximum 3.23 (− 13.39–18.45)	Minimum 0.005 maximum 0.001	Minimum 0.03 maximum 0.03

Group A (6–8 years), Group B (9–10 years), Group C (11–12 years).

Differences between the three groups were analyzed using the Kruskal–Wallis test or one-way analysis of variance. Variables with significant differences were subsequently compared using multiple comparison analyses with Bonferroni correction. Data are presented as means (standard deviation) or median values (range). *P* values < 0.0001 were considered statistically significant.

**Table 5:** The minimum and maximum values for the gait kinematics of the knee, ankle and foot progression angle for the three age groups (N = 424).

Variables	Group A (n = 141)	Group B (n = 154)	Group C (n = 129)	*P* value	Effect size (η^2^)
Knee flexion/extension angle (degrees)	Stance phase minimum 2.12 (4.38) swing phase maximum 60.75 (4.77)	Stance phase minimum 3.53 (3.48) swing phase maximum 61.03 (4.51)	Stance phase minimum 2.68 (3.49) swing phase maximum 57.95 (4.40)	Stance phase minimum 0.006 swing phase maximum A and B: 1.000 A and C: < 0.0001 B and C: < 0.0001	Stance phase minimum 0.02 swing phase maximum 0.08
Knee flexion/extension ROM of gait cycle (degrees)	60.11 (5.19)	58.98 (4.16)	56.90 (5.00)	A and B: 0.129 A and C: < 0.0001 B and C: < 0.001	0.07
Ankle dorsiflexion/plantarflexion swing phase minimum angle (degrees)	− 17.49 (− 33.96–1.56)	− 17.16 (− 33.24 to − 1.38)	− 18.00 (− 32.09 to − 3.76)	0.433	0.004
Ankle dorsiflexion/plantarflexion stance phase maximum angle (degrees)	15.15 (3.89)	14.43 (3.49)	13.88 (3.33)	0.014	0.02
Foot progression internal/external rotation stance phase angle (degrees)	Minimum − 6.41 (5.75) maximum 0.14 (6.03)	Minimum − 6.08 (5.88) maximum 0.16 (6.31)	Minimum − 5.81 (4.94) maximum 0.11 (5.32)	Minimum 0.674 maximum 0.997	Minimum 0.002 maximum 0.001
Foot progression internal/external rotation ROM of gait cycle (degrees)	15.54 (7.44–30.15)	13.84 (5.61–25.34)	14.63 (5.87–28.81)	A and B: < 0.0001 A and C: 0.003 B and C: 1.000	0.04

Group A (6–8 years), Group B (9–10 years), Group C (11–12 years).

Differences between the three groups were analyzed using the Kruskal–Wallis test or one-way analysis of variance. Variables with significant differences were subsequently compared using multiple comparison analyses with Bonferroni correction. Data are presented as means (standard deviation) or median values (range). *P* values < 0.0001 were considered statistically significant.

ROM, range of motion.

**Table 6:** The minimum and maximum values for the kinetics of the lower extremity for the three age groups (n = 424).

Variables	Group A (n = 141)	Group B (n = 154)	Group C (n = 129)	*P* value	Effect size (η^2^)
Sagittal hip minimum moment (Nm/Kg)	− 0.61 (− 1.01 to − 0.21)	− 0.64 (− 1.10 to − 0.32)	− 0.63 (− 1.11 to − 0.31)	0.096	0.01
Sagittal knee maximum moment (Nm/Kg)	0.44 (0.06–0.87)	0.51 (0.16–1.43)	0.52 (0.06–0.99)	0.001	0.03
Sagittal ankle maximum moment (Nm/Kg)	1.02 (0.13)	1.17 (0.16)	1.25 (0.14)	A and B: < 0.0001	0.3
				A and C: < 0.0001	
				B and C: < 0.0001	
Sagittal hip maximum power (W/Kg)	0.73 (0.31–1.70)	0.86 (0.35–2.36)	0.84 (0.39–2.09)	A and B: 0.002	0.04
				A and C: 0.002	
				B and C: 1.000	
Sagittal knee minimum power (W/Kg)	− 0.51 (− 2.14 to − 0.13)	− 0.56 (− 2.35 to − 0.01)	− 0.60 (− 1.74–0.15)	0.269	0.01
Sagittal ankle maximum power (W/Kg)	2.57 (0.56–4.81)	2.92 (1.22–5.44)	2.76 (1.10–4.80)	0.003	0.03

Group A (6–8 years), Group B (9–10 years), Group C (11–12 years).

The moment values denote the minimum or maximum moment during the gait cycle. The power values denote the minimum or maximum power over the gait cycle. Units: moment: Nm/kg; power: W/kg. Negative values for hip moments indicate flexion moments. Negative values for power indicate absorption.

Differences between the three groups were analyzed using the Kruskal–Wallis test or one-way analysis of variance. Variables with significant differences were subsequently compared using multiple comparison analyses with Bonferroni correction. Data are presented as means (standard deviation) or median values (range). *P* values < 0.0001 were considered statistically significant.

In addition, there was a statistically significant difference in the range of knee angle motion during the gait cycle between Groups A and C, with a greater range of knee angle motion in Group A than in Group C. Moreover, there was a statistically significant difference in the foot progression angle range of motion during the gait cycle between Groups A and B; Group A had a greater foot progression range of motion than Group B. There were statistically significant differences in the sagittal ankle maximum moment between all groups; the post hoc comparisons revealed that Group C had a greater sagittal ankle moment than Groups A and B, and Group B had a greater sagittal ankle moment than Group A (Table 6). There were no statistically significant between-group differences in other spatiotemporal parameters, kinematic, and kinetic parameters (Supplementary Tables S1 and S2 online).

**Figure 1:**
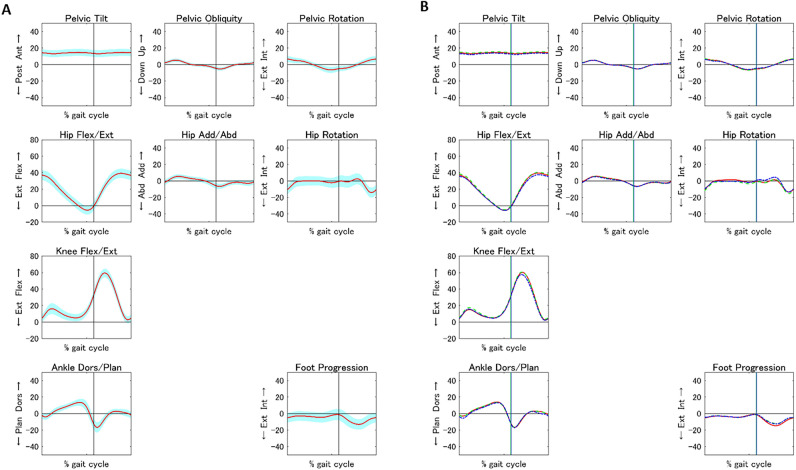
*Mean kinematic curve generated using the values of the following gait standards in the total population and three subgroups: pelvis tilt, pelvis obliquity, pelvis rotation, hip flexion/extension, hip abduction/adduction, hip rotation, knee flexion/extension, ankle dorsiflexion/plantarflexion, foot progression*. (**a**) Mean kinematic curve (red line) with the standard deviation (blue area) in the whole population. (**b**) Mean kinematic curves in the three age groups, including Groups A (6–8 years, red line), B (9–10 years, green dotted line), and C (11–12 years, blue dotted line). Abd, abduction; Add, adduction; Ant, anterior; Dors, dorsiflexion; Ext, extension or external; Flex, flexion; Int, internal; Plan, plantarflexion; Post, posterior.

**Figure 2:**
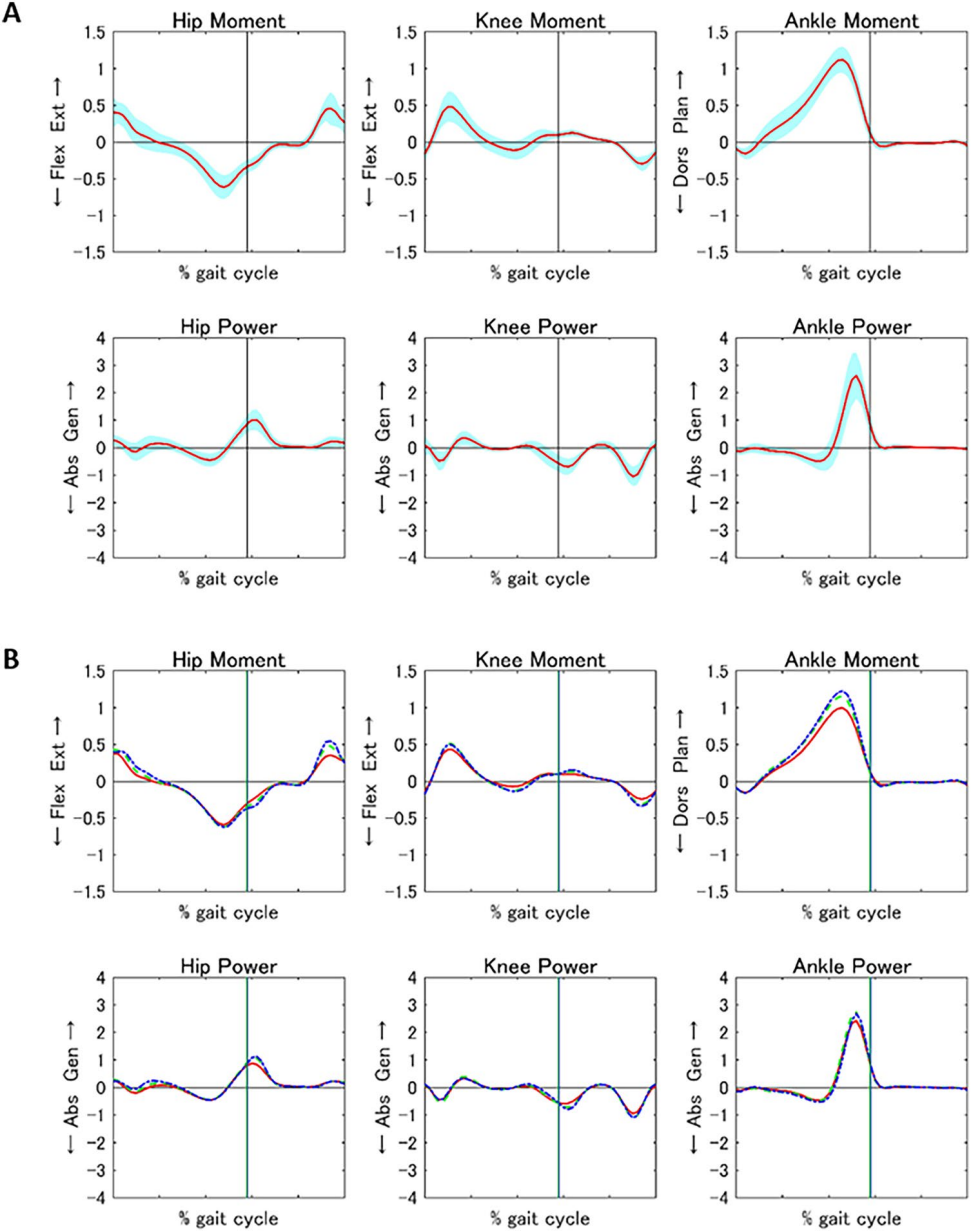
*Mean kinetic curve generated using the values of the gait standards in the total population and three subgroups: hip flexion/extension, hip knee flexion/extension, ankle dorsiflexion/plantarflexion, hip power, knee power, and ankle power*. (**a**) Mean kinetic curve (red line) with the standard deviation (blue area) in the whole population. (**b**) Mean kinetic curves in the three age groups, including Groups A (6–8 years, red line), B (9–10 years, green dotted line), and C (11–12 years, blue dotted line). Abs, absorption; Dors, dorsiflexion; Ext, extension; Flex, flexion; Gen, generation; Plan, plantarflexion.

**Figure 3:**
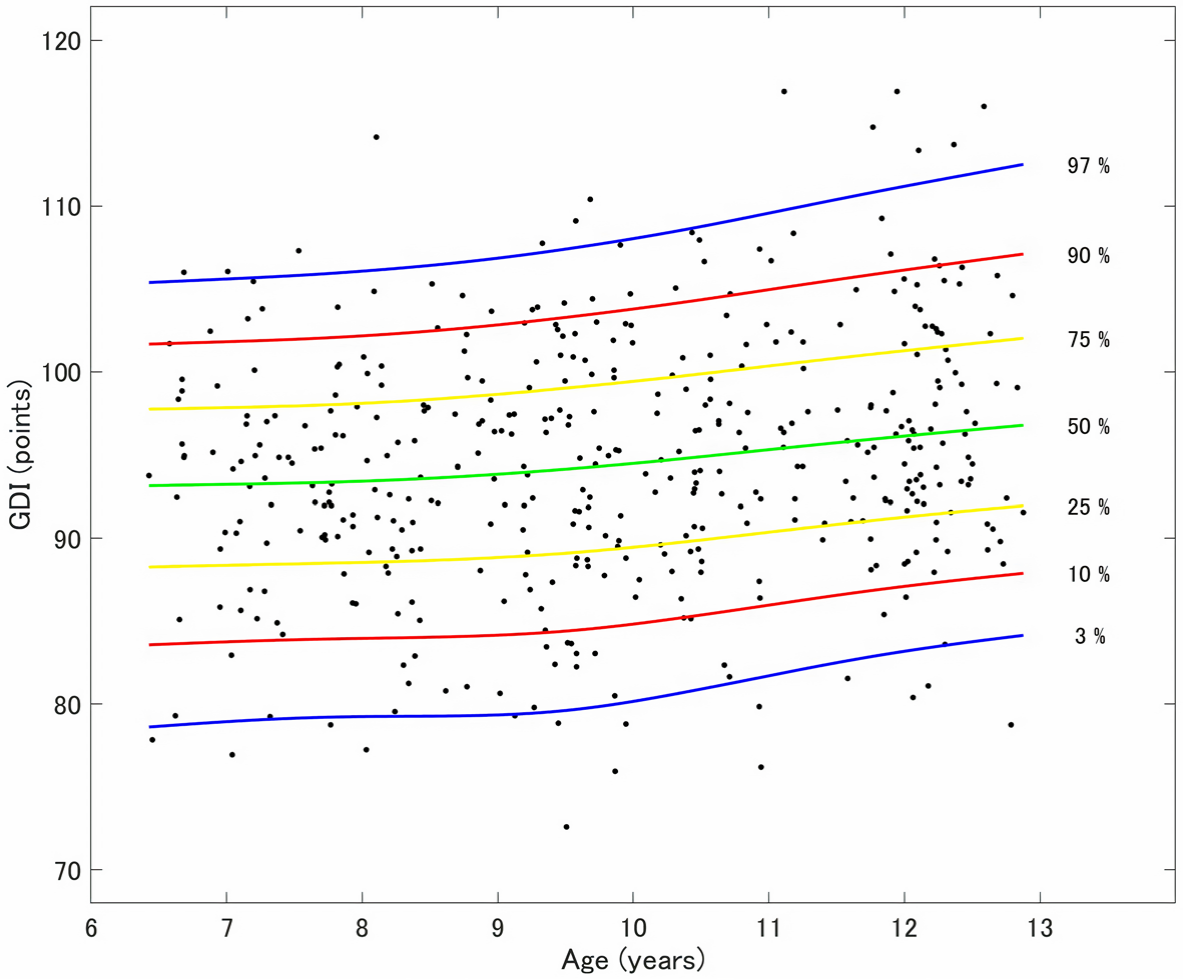
*Centile chart of the Gait Deviation Index (GDI) standards by age*. The developmental centile chart of the gait pattern was created using LMS Chartmaker, version 2.54 (Medical Research Council, London, UK). The 97th percentile represents the 97th value obtained after arranging the GDI data from the lowest to the highest values among 100 children of the same age; similarly, the 3rd percentile represents the third GDI value after arranging data from the lowest to the highest values. From top to bottom, the seven reference lines depict the 97th (blue), 90th (red), 75th (yellow), 50th (green), 25th (yellow),10th (red), and 3rd (blue) percentile curves. The color of each line corresponds to the number of standard deviations (SD) for each percentile shown (3rd percentile: − 1.88 SD; 10th percentile: − 1.28SD; 25th percentile: − 0.67SD; 50th percentile: median; 75th percentile: 0.67SD; 90th percentile: 1.28SD; and 97th percentile: 1.88SD).

We constructed mean kinematic and kinetic curves for clinical application using the standard gait values in the total population and three subgroups (Figs. 1 and 2), and we created a centile chart of the GDI standards by age (Fig. 3).

## Discussion

To our knowledge, this is the first study to determine the normative gait pattern characteristics of Japanese children exhibiting normal development. Studies on changes in gait patterns and parameters over time and with age have been performed in Australia, China, Germany, and the USA^12,13,17,26,27^. The results of this study indicate that gait patterns and gait parameters of typical Japanese children aged 6–12 years are similar to those reported in the published international literature on the gait patterns of children in developed countries^12,13,17,21–23,26–28^. In addition, developmental centile charts of gait patterns clearly describe typical changes in gait quality throughout childhood while accommodating the observed variations in gait patterns as children develop.

We used three age groups to assess the change in gait parameters related to growth and development. Previous studies based on non-normalized data reported a faster mean cadence and longer step and stride lengths in older children than those in younger children^6,8,14^. The results of this study are consistent with previous reports showing that spatiotemporal parameters change as children mature^8,27,29^. In contrast with the findings of this study, previous studies reported no significant differences in the normalized cadence or the step and stride lengths by age among children^8,13,15,16,30^. The results of this study revealed an increase in cadence among children in late elementary school grades.

Moreover, step and stride lengths decreased with age when children aged 11–12 years were compared with those aged 6–8 years; however, no age-related change was observed in the normalized gait speed. The normalization results suggest that increased cadence compensated for the decreases in step and stride lengths to maintain gait speed in those with late elementary school ages.

Step, stride, and single support times in this study were shortened in those with ages corresponding to late elementary grades. Noel et al. reported that the normalized single support time and step time in children were unaffected by age^12,13^, which is inconsistent with our present findings. These discrepancies may have resulted from variability in participant characteristics, such as differences in age groups and physiques, and differences in the definition and classification of the inclusion criteria and gait parameter measurement methods.

The kinematic patterns were similar among children aged 6–12 years. This could be attributed to negligible differences in the normalized gait speed and the absence of gross structural or developmental abnormalities in the participants^29^. Smith et al.^8^ and Ciğali et al.^18^ also reported mean peak values for knee flexion/extension, ankle dorsiflexion/plantarflexion, and foot progression angle, all of which fell within the standard deviation of our study (Fig. 1).

Our findings suggest that, in developing children, sagittal movements of the hip and knee gradually decrease in late elementary grades; however, there is less change in the movement of the ankle joint. At the minimum angle of the stance phase, the hip and knee exhibited a similar position in the sagittal plane for all age groups. However, as the gait progressed, children aged 11–12 years had less maximum knee flexion movement during the swing phase and less range of knee movement during the gait cycle. This might have been because children aged 11–12 years have shorter normalized step and stride lengths. The longer normalized step and stride lengths might be a compensatory mechanism for the excessive hip and knee maximum flexion angles in the stance and swing phases, respectively, in children aged 9–10 years. At the minimum and maximum angles of the stance phase, the foot progression angle had a similar rotational position in the horizontal plane for all age groups. However, children aged 9–10 years demonstrated less overall movement of the internal/external foot progression angle during the gait cycle than those aged 6–8 years. Whether changes in kinematic performance on the foot progression angle were unique to the participants of this study and whether these kinematic data are clinically meaningful requires further study.

The main differences in the joint moment between children aged 6–12 years were observed in the ankle joints. In this study, children aged 11–12 years had a higher plantarflexion moment in the ankle’s sagittal movements than those aged 6–8 and 9–10 years. Fukuchi et al.^31^ reported on joint kinetics and observed large effect sizes for the hip flexion and knee extension moments, with a moderate effect size for the ankle plantarflexion moments. Additionally, the findings in the three groups in this study are similar to those of previous studies using plantar pressure measurements that reported a gradual increase in peak pressure during typical childhood development^32,33^.

Previous studies have reported the walking speed dependencies for the ankle moment^34–36^. The results of this study are consistent with those of previous studies and suggest that as a child develops, the increase in gait speed causes older children to walk with a higher ankle moment during push-off than younger children. The power of each joint did not change significantly with age. Ounpuu et al.^20^ also reported mean peak values for hip and knee flexion/extension and ankle dorsiflexion/plantarflexion power, which fall within the standard deviation of those measurements in our study (Fig. 2). Thus, joint power during gait in Japanese children aged 6–12 years falls within the international norms^20,28,37,38^.

Age-continuous analysis of GDI scores standards describe typical changes in gait patterns in children aged 6–12 years, while accommodating the observed variation in GDI scores as children develop. This analysis builds on a previous study that reported developmental centile charts of gait parameter standards^21^, and, to our knowledge, is the first study to construct a developmental centile chart of gait patterns. In addition, this study had the largest sample size among studies analyzing GDI scores according to age-related standards. Alderson et al.^21^ reported that the measurement of centiles allows for the comparison of performance using standard values. This developmental centile chart of gait pattern standards can facilitate the identification of specific deviations from a typical developmental trajectory in clinical settings when used in conjunction with the mean kinematic and kinetic curves created in this study. The developmental centile charts of gait patterns could also easily be combined with performance measurements, such as physical function measurement. Future studies should consider including this combination in the methodological design.

Among typically-developed 6–10-year-old children in South Africa, older children exhibited a greater hip external rotation angle than younger children^8^. Meanwhile, among French children aged 6–12 years, maturation did not occur by the age of 12, and their gait pattern differs from that of children from other countries, even when normalized^22^. Moreover, during late stance in children from the United States of America, 7-year-olds presented with less peak ankle power absorption and generation and diminished peak plantar flexor moments at the ankle^23^. Previous studies reported no significant differences in the normalized cadence and step and stride lengths among children classified according to age^8,13,15,16^. However, the results of this study revealed an increase in cadence among children in the 11–12-year-old group compared with that in the 6–8-year-olds. Also, the results of this study revealed a decrease in the step and stride length among children aged 11–12 years compared with the measurements in those aged 9–10 years. Meanwhile, previous studies reported that kinematic gait patterns were similar between younger and older children^8^, and this study showed as the gait progressed, those aged 11–12 years had a lower maximum knee flexion movement during the swing phase and less range of motion of the knee during the gait cycle. Furthermore, in this study, a higher plantarflexion moment in the ankle’s sagittal movements was observed as children aged; however, maintaining the maximum internal rotation angle did not significantly alter the hip rotation movements as children aged, a finding that differed from those of studies involving South African children. Hence, the gait kinematics and kinetics of Japanese children aged 6–12 years differ from those reported in children from other countries. Age-related changes in the cadence and step and stride lengths appear to be similar worldwide; however, the normalized values slightly differ from those in our study. The age-related centile chart of GDI standards provides a simple means of assessing a child’s current gait pattern. Our 3D gait analysis database may be of value in future gait studies involving children.

This study provides information that will allow future studies to overcome some common limitations. In order to obtain more reliable gait standards for Japanese children; it may be necessary to include participants from other age groups and to make comparisons according to age and with data from other regions.

### Clinical messages

- Children’s gait kinematics and kinetics differ slightly by country.
- Older children had a higher maximum ankle moment than that of younger children.
- Cadence and step and stride lengths change with age.

## Methods

### Study population

Between January 2018 and March 2020, students from two elementary schools in Okazaki City were invited for an Okazaki child medical check-up to assess physical function at our medical center. Participants were required to have no orthopedic or neurological abnormalities and to have Raven’s Colored Progressive Matrices and Picture Vocabulary Test-Revised scores^39,40^ within the normal range. Children with acoustic, cardiovascular, neurological, ophthalmologic, and orthopedic abnormalities, those with the inability to complete gait analysis, and individuals with autism spectrum disorder and attention deficit hyperactivity disorder diagnoses were excluded.

The study was conducted in accordance with the Declaration of Helsinki, and the protocol was approved by the Ethics Committee of the Aichi Prefectural Mikawa Aoitori Ethics Review Board (approval number: 29002). The manuscript was prepared according to the Strengthening the Reporting of Observational Studies in Epidemiology (STROBE) guidelines. The study was conducted in accordance with the Health Insurance Portability and Accountability Act of 1996 (HIPAA) Privacy Rule. The legal guardians of all participants provided written informed consent for participation in the study and for the publication of identifying information and images.

### Data collection

Measurements were performed using an eight-camera motion analysis system at a sampling frequency of 100 Hz (MX-T 20S; Vicon, Oxford, UK). Instrumented 3D gait analysis was performed using the Plug-In-Gait model to obtain lower extremity kinematics at the pelvis, hip, knee, and ankle of both sides. 8-AMTI OPT force plates (Advanced Mechanical Technology, Inc., Watertown, MA, USA) were used to evaluate the lower extremity kinetics at the hip, knee, and ankle of both sides. After collecting anthropometric data, participants were equipped with 16 retro-reflective markers (Plug-In-Gait lower body Ai; Vicon, Oxford, UK) by physiotherapists experienced in clinical gait analysis. The Plug-In-Gait lower body Ai marker set was placed on the anterior and posterior superior iliac spines, lateral femur, lateral knee joint, lateral lower leg, lateral malleolus of the ankle joint, head of the second metatarsal, and calcaneus on both sides. Following a static measurement trial conducted in the upright position and used to indicate the start and the end of the path, an approach of at least 3 m was allowed before reaching the force plate so that participants could attain their usual gait pace before reaching the timed path. They were instructed to continue their gait past the end of the force plate for an additional 3 m to ensure that the gait pace was consistent throughout the task, and at least three trials were performed and subsequently recorded^41–44^. The kinematics and kinetics data from the three trials for each participant were exported from Polygon (Vicon Polygon 4.4.2; Vicon, Oxford, UK) to Excel (Microsoft, Redmond, WA, USA). The means for the kinematic and kinetic variables were calculated over three gait analysis trials for the right and left legs.

### GDI

The participants’ GDI values were calculated using the current Vicon pipeline as previously described^45^. The GDI represents overall gait patterns using numerical values and was developed using kinematic data from the pelvis, hip, knee, ankle, and foot progression angles as an average value of several gait cycles. Kinematic data were incorporated from the pelvis, hip, knee, and ankle joints; the pelvis and hip joints; and the pelvis, hip, and foot progression in the sagittal, frontal, and transversal planes, respectively. The GDI has been revealed to have high validity, with excellent inter-trial and intra-rater reliability^45^. A total of 459 kinematic data points, and nine angular kinematic variables were captured 51 times (every 2% of the gait cycle). The mean GDI over the three trials was calculated for each leg. Higher and lower GDI values are indicative of better and poorer overall joint motion of the lower extremity during gait, respectively.

### Spatiotemporal parameters

The cadence, stride time, opposite foot off, opposite foot contact, step time, single support, double support, foot off, stride length, and step length were calculated. The following gait parameters were determined: cadence, the number of steps per minute; stride time, the time between the first toe off and the last foot contact of each stride; opposite foot off, the proportion of the gait cycle (stride time) in which the opposite foot off event occurs; opposite foot contact, the proportion of the gait cycle (stride time) in which the opposite foot contact event occurs; step time, the time between toe off and initial contact in swing phase; single support, the duration (s) for which only one foot is down; double support, the proportion of the stride time in which both feet are in contact with the floor during that stride (between the first foot contact and the second toe off); foot off, the proportion of the gait cycle (stride time) in which the foot off event occurs; stride length, the global distance between the position of the anterior foot marker (typically the left or right toe) between foot contact events at the beginning and end of a cycle; and step length, the anterior–posterior distance between the head of the second metatarsal and the calcaneus foot marker at each initial contact.

Gait speed was recorded in m/s. The mean of each gait parameter was calculated over the three trials for each leg. The spatiotemporal parameters were normalized using leg length, according to the following formulae: step length (m) = step length/leg length; stride length (m) = stride length/leg length; cadence (steps/min) = cadence × SQRT (leg length/*g*); gait speed (m/s) = speed/SQRT (leg length × *g*); and step time (s), stride time (s), single support (s), and double support (s) = seconds/SQRT (leg length/*g*), where *g* refers to the acceleration due to gravity (9.81 ms^−2^)^46^.

### Developmental centile charts of gait pattern

Developmental centile charts of gait patterns were created using LMS Chartmaker, version 2.54 (Medical Research Council, London, UK)^47^. The LMS method is a fitting process that changes the distribution by three variables: L (Box-Cox transformation), M (median), and S (coefficient of variation)^48^. These three curves can be fitted as cubic splines by non-linear regression using penalized likelihood, and smoothed centile curves can be estimated using the chosen equivalent degrees of freedom (edf) for the L, M, and S curves^48^. The equation used to derive the centile is as follows:

$$ {\text{Centile}}\left( {\text{t}} \right)\, = \,{\text{M}}\left( {\text{t}} \right)\left[ {{1}\, + \,{\text{L}}\left( {\text{t}} \right){\text{S}}\left( {\text{t}} \right){\text{Z}}\left( {\text{t}} \right)} \right]^{{{1}/{\text{L}}({\text{t}})}} $$

where L, M, and S are age-specific values, and Z is the value of a given percentile in the cumulative standard normal distribution. The goodness-of-fit of the model was checked using Q-tests and subsequently improved by adjusting the edf for the L, M, and S curves, if necessary.

### Statistical analysis

All statistical analyses were performed using IBM SPSS Statistics software, version 24.0 (IBM Corp., Armonk, N.Y., USA). Kinematic and kinetic data were acquired using a sampling frequency of 100 Hz and calculated using MATLAB (MathWorks Inc., Natick, MA, USA). Effect sizes were calculated using *r* or Cramer's V. Effect sizes with η^2^ = 0.01 or η^2^ =  − 0.01 were considered small, those with η^2^ = 0.06 or η^2^ =  − 0.06 were moderate, and those with η^2^ = 0.14 or η^2^ =  − 0.14 were large. A power analysis was conducted with G*Power (Heinrich Heine University, Düsseldorf, Germany) using an alpha of 0.0001, a power of 0.8, and a medium effect size (f = 0.25)^49,50^. Based on these assumptions, the required sample size was determined to be 414. The three age groups were categorized as follows: Group A, 6–8 years; Group B, 9–10 years; and Group C, 11–12 years.

The Vicon Plug-In-Gait model yielded estimates for the sagittal, frontal, and transverse positions of the hip, knee, ankle, foot progression, and pelvis over the observed right and left gait cycles. We calculated the mean values of the sagittal, frontal, and transverse movements of the hip, knee, ankle, foot progression, and pelvis (averaged over all participants and both left and right gait cycles) for percentages of the observed gait cycles in the three age groups (6–8 years, 9–10 years, and 11–12 years). Gap filling was performed using a standard Woltring filter supplied by Vicon. Descriptive statistics (median, range) were used to describe participant demographics, and medians and ranges or means and standard deviations were used to describe the outcome measures. The normal distribution of each variable was confirmed using the Shapiro–Wilk test. The Chi-square test was used to compare differences in proportions according to sex in each group. Differences in gait kinematics, kinetics, GDI, and spatiotemporal parameters between the three groups were analyzed using a one-way analysis of variance or the Kruskal–Wallis test. Variables with significant differences were subsequently compared using multiple comparison analyses with Bonferroni correction. *P* values < 0.0001 were considered statistically significant.

We confirmed that the children showed no significant sex-based differences in the GDI values (Supplementary Table S3 online). A previous study reported that gait parameters in children change according to their height, at a breakpoint, and after reaching 120 cm in height^22^. In order to explore the results in detail, the children were divided into three groups classified according to body height (100–120 cm, 121–140 cm, and ≥ 141 cm) (Supplementary Table S4 online). The results of this study indicate that the gait patterns and gait parameters of typical Japanese children classified according to body height are similar to those observed when children were classified according to age (Supplementary Tables S5, S6, S7, S8, S9, S10 online). Meanwhile, the results differed only in terms of the hip flexion/extension range of motion of the gait cycle and opposite foot off parameters, and the GDI.

## Supplementary Information

Supplementary Information 1.

Supplementary Information 2.

## Data Availability

All the relevant data are presented in the manuscript. All data are available from the authors upon request.
