# Supplementary material for: Three-dimensional gait analysis of lower extremity gait parameters in Japanese children aged 6 to 12 years
Source: Sci Rep. 2022 May 12;12:7822. doi: 10.1038/s41598-022-11906-1 (PMC9098504; doi:10.1038/s41598-022-11906-1)
Supplement: Supplementary file 2 — Supplementary Information 2. [file 41598_2022_11906_MOESM2_ESM.docx]

**Supplementary Information**

**Three-Dimensional Gait Analysis of Lower Extremity Gait Parameters in Japanese Children Aged 6 to 12 Years**

Tadashi Ito^1,2,*^, Koji Noritake^3^, Yuji Ito^4,5^, Hidehito Tomita^6,7^, Jun Mizusawa^7^, Hiroshi Sugiura^3^, Naomichi Matsunaga^2^, Nobuhiko Ochi^5^, Hideshi Sugiura^2^

^1^ Three-Dimensional Motion Analysis Room, Aichi Prefectural Mikawa Aoitori Medical and Rehabilitation Center for Developmental Disabilities, Okazaki, Japan

^2^ Department of Integrated Health Sciences, Graduate School of Medicine, Nagoya University, Nagoya, Japan

^3^ Department of Orthopedic Surgery, Aichi Prefectural Mikawa Aoitori Medical and Rehabilitation Center for Developmental Disabilities, Okazaki, Japan

^4^ Department of Pediatrics, Nagoya University Graduate School of Medicine, Nagoya, Japan

^5^ Department of Pediatrics, Aichi Prefectural Mikawa Aoitori Medical and Rehabilitation Center for Developmental Disabilities, Okazaki, Japan

^6^ Graduate School of Health Sciences, Toyohashi SOZO University, Toyohashi, Japan

^7^ Department of Rehabilitation, Aichi Prefectural Mikawa Aoitori Medical and Rehabilitation Center for Developmental Disabilities, Okazaki, Japan

***Corresponding author**

Tadashi Ito, PT, PhD

Three-Dimensional Motion Analysis Room, Aichi Prefectural Mikawa Aoitori Medical and Rehabilitation Center for Developmental Disabilities, 9-3 Koyaba Kouryuji cho, 444-0002, Okazaki, Japan

Phone: +81-564-64-7980, Fax: +81-564-64-7981, E-mail: sanjigen@mikawa-aoitori.jp

**Table S1.** Temporal parameters, and minimum and maximum values for the gait kinematics of the pelvis and lower extremity for the three age groups (N = 424)

| **Variables** | **Group A**  **(n = 141)** | **Group B**  **(n = 154)** | **Group C**  **(n = 129)** | ***P*-value** | **Effect size (η^2^)** |
| --- | --- | --- | --- | --- | --- |
| Temporal parameters |  |  |  |  |  |
| Foot off (%) | 57.78 (1.37) | 57.75 (1.17) | 58.16 (1.23) | 0.013 | 0.02 |
| Opposite foot off (%) | 7.56 (4.51–21.30) | 7.85 (4.63–10.87) | 8.33 (5.54–12.31) | 0.001 | 0.03 |
| Opposite foot contact (%) | 50.01 (48.90–52.10) | 49.91 (47.78–51.09) | 49.89 (49.18–51.05) | 0.029 | 0.02 |
| Kinematics |  |  |  |  |  |
| Pelvis tilt anterior/posterior ROM of gait cycle (degrees) | 3.36 (1.24–9.20) | 3.35 (1.31–6.71) | 3.15 (0.96–6.60) | 0.842 | 0.001 |
| Pelvis obliquity upward/downward ROM of gait cycle (degrees) | 10.52 (4.47–18.11) | 10.98 (4.00–19.34) | 10.35 (5.65–19.18) | 0.232 | 0.01 |
| Pelvis Rotation internal /external rotation ROM of one gait cycle (degrees) | 14.37 (3.56–32.18) | 14.21 (4.26–39.94) | 12.89 (3.62–30.40) | 0.052 | 0.01 |
| Hip flexion/extension ROM of gait cycle (degrees) | 46.28 (5.32) | 46.34 (4.83) | 44.14 (4.91) | A and B: < 1.000  A and C: < 0.002  B and C: < 0.001 | 0.04 |
| Hip adduction/abduction ROM of gait cycle (degrees) | 12.83 (5.74–19.23) | 13.01 (6.28–19.95) | 12.49 (7.20–18.84) | 0.186 | 0.01 |
| Hip internal /external rotation ROM of gait cycle (degrees) | 19.24 (10.53–31.31) | 19.60 (10.74–48.79) | 19.82 (10.78–35.18) | 0.739 | 0.001 |
| Ankle dorsiflexion/plantarflexion ROM of gait cycle (degrees) | 32.77 (5.97) | 31.86 (5.10) | 32.07 (5.26) | 0.336 | 0.01 |

Group A (6–8 years), Group B (9–10 years), Group C (11–12 years)

Differences between the three groups were analyzed using the Kruskal–Wallis test or one-way analysis of variance depending on the distribution of the data. Variables with significant differences were subsequently compared using multiple comparison analyses with Bonferroni correction. Data are presented as means (standard deviation) or median values (range). *P*-values < 0.0001 were considered statistically significant.

ROM, range of motion

**Table S2.** The values for the power at integral in the gait cycle for the three age groups (N = 424)

| **Variables** | **Group A**  **(n = 141)** | **Group B**  **(n = 154)** | **Group C**  **(n = 129)** | ***P*-value** | **Effect size (η^2^)** |
| --- | --- | --- | --- | --- | --- |
| Sagittal hip power at integral in gait cycle (Nm s/Kg) | 14.28 (7.26–26.82) | 15.50 (6.43–35.58) | 14.97 (8.19–29.47) | 0.036 | 0.02 |
| Sagittal knee power at integral in gait cycle (Nm s/Kg) | 14.34 (5.04–28.07) | 16.01 (7.19–50.10) | 15.55 (7.78–33.64) | 0.002 | 0.03 |
| Sagittal ankle power at integral in gait cycle (Nm s/Kg) | 19.57 (6.74–31.38) | 21.39 (9.17–37.54) | 20.36 (10.45–34.38) | 0.003 | 0.03 |

Group A (6–8 years), Group B (9–10 years), Group C (11–12 years)

The power values denote the integral power over the gait cycle.

Differences between the three groups were analyzed using the Kruskal–Wallis test. Data are presented as median values (range). *P*-values < 0.0001 were considered statistically significant.

**Table S3.** Gait Deviation Index for each sex and age group (N = 424)

| **Variable** | **Group A girls**  **(n = 72)** | **Group A boys**  **(n = 69)** | **Group B girls**  **(n = 81)** | **Group B boys**  **(n = 73)** | **Group C girls**  **(n = 63)** | **Group C boys**  **(n = 66)** | ***P*-value** | **Effect size (η^2^)** |
| --- | --- | --- | --- | --- | --- | --- | --- | --- |
| Gait Deviation Index (points) | 93.68 (6.46) | 93.04 (7.40) | 93.73 (7.23) | 93.93 (8.31) | 97.52 (7.46) | 96.16 (7.25) | 0.002 | 0.04 |

Group A (6–8 years), Group B (9–10 years), Group C (11–12 years)

Differences between the three groups were analyzed by one-way analysis of variance. Data are presented as the means (standard deviations). *P*-values < 0.0001 were considered statistically significant.

There was no significant between-group difference for GDI in terms of sex.

**Table S4.** Demographic characteristics of the participants for each body height classification (N = 424)

| **Variables** | **Group 1, 100–120 cm**  **(n = 57)** | **Group 2, 121–140 cm**  **(n = 226)** | **Group 3, ≥ 141 cm**  **(n = 141)** | ***P*-value** | **Effect size (η^2^) or Cramer V** |
| --- | --- | --- | --- | --- | --- |
| Age (years) | 7.0 (6–9) | 9.0 (6–12) | 11.0 (9–12) | 1 and 2: < 0.0001  1 and 3: < 0.0001  2 and 3: < 0.0001 | 0.63 |
| Sex, n (%) |  |  |  | 0.838 | 0.03 |
| Boys | 27 (47.4) | 109 (48.2) | 72 (49.1) |  |  |
| Girls | 30 (52.6) | 117 (51.8) | 69 (49.1) |  |  |
| Height (cm) | 116.7 (106.5–120.9) | 130.1 (121.1–140.9) | 147.8 (141.1–164.2) | 1 and 2: < 0.0001  1 and 3: < 0.0001  2 and 3: < 0.0001 | 0.81 |
| Weight (kg) | 19.8 (16.1–26.0) | 26.2 (19.2–44.7) | 37.4 (26.3–74.4) | 1 and 2: < 0.0001  1 and 3: < 0.0001  2 and 3: < 0.0001 | 0.64 |
| Body mass index (kg/m^2^) | 14.55 (12.98–18.14) | 15.43 (12.91–26.62) | 16.90 (12.32–29.60) | 1 and 2: < 0.0001  1 and 3: < 0.0001  2 and 3: < 0.0001 | 0.17 |

Differences between the three groups were analyzed using the Kruskal–Wallis test or one-way analysis of variance depending on the distribution of the data. Variables with significant differences were subsequently compared using multiple comparison analyses with Bonferroni correction. Data are presented as means (standard deviation) or median values (range). P-values < 0.0001 were considered statistically significant.

**Table S5.** Temporal parameters for each body height classification (N = 424)

| **Variables** | **Group 1, 100–120 cm**  **(n = 57)** | **Group 2, 121–140 cm**  **(n = 226)** | **Group 3, ≥ 141 cm**  **(n = 141)** | ***P*-value** | **Effect size (η^2^)** |
| --- | --- | --- | --- | --- | --- |
| Cadence (steps/min) | 130.34 (115.54–164.82) | 128.35 (93.50–164.51) | 121.18 (91.06–151.19) | 1 and 2: 0.053  1 and 3: < 0.0001  2 and 3: < 0.0001 | 0.16 |
| Gait speed (m/s) | 1.05 (0.83–1.57) | 1.17 (0.65–1.75) | 1.24 (0.84–1.77) | 1 and 2: < 0.001  1 and 3: < 0.0001  2 and 3: 0.005 | 0.08 |
| Step time (s) | 0.45 (0.35–0.53) | 0.47 (0.36–0.64) | 0.50 (0.40–0.66) | 1 and 2: 0.044  1 and 3: < 0.0001  2 and 3: < 0.0001 | 0.17 |
| Stride time (s) | 0.90 (0.74–1.04) | 0.94 (0.73–1.29) | 0.99 (0.80–1.32) | 1 and 2: 0.054  1 and 3: < 0.0001  2 and 3: < 0.0001 | 0.16 |
| Single support (s) | 0.38 (0.23–0.45) | 0.40 (0.32–0.50) | 0.42 (0.19–0.53) | 1 and 2: < 0.016  1 and 3: < 0.0001  2 and 3: < 0.0001 | 0.14 |
| Double support (s) | 0.14 (0.07–0.21) | 0.15 (0.08–0.30) | 0.16 (0.06–0.27) | 1 and 2: < 0.791  1 and 3: < 0.0001  2 and 3: < 0.0001 | 0.08 |
| Foot off (%) | 57.31 (53.94–60.59) | 57.76 (54.31–62.73) | 58.27 (55.22–62.67) | 0.001 | 0.02 |
| Opposite foot off (%) | 7.34 (4.51–21.30) | 7.79 (4.89–11.67) | 8.37 (4.63–12.31) | 1 and 2: 1.000  1 and 3: 0.002  2 and 3: < 0.0001 | 0.04 |
| Opposite foot contact (%) | 50.01 (49.01–52.10) | 49.96 (47.78–51.05) | 49.88 (49.00–51.09) | 0.062 | 0.01 |

Differences between the three groups were analyzed using the Kruskal–Wallis test of variance depending on the distribution of the data. Variables with significant differences were subsequently compared using multiple comparison analyses with Bonferroni correction. Data are presented as median values (range). *P*-values < 0.0001 were considered statistically significant.

**Table S6.** Spatial parameters and Gait Deviation Index for each body height classification (N = 424)

| **Variables** | **Group 1, 100–120 cm**  **(n = 57)** | **Group 2, 121–140 cm**  **(n = 226)** | **Group 3, ≥ 141 cm**  **(n = 141)** | ***P*-value** | **Effect size (η^2^)** |
| --- | --- | --- | --- | --- | --- |
| Step length (m) | 0.48 (0.06) | 0.55 (0.06) | 0.61 (0.06) | 1 and 2: < 0.0001  1 and 3: < 0.0001  2 and 3: < 0.0001 | 0.32 |
| Stride length (m) | 0.96 (0.11) | 1.09 (0.12) | 1.21 (0.12) | 1 and 2: < 0.0001  1 and 3: < 0.0001  2 and 3: < 0.0001 | 0.32 |
| Gait Deviation Index (points) | 93.75 (76.95–106.05) | 94.18 (72.60–114.75) | 95.45 (78.75–116.9) | 0.011 | 0.02 |

Differences between the three groups were analyzed using the Kruskal–Wallis test or one-way analysis of variance depending on the distribution of the data. Variables with significant differences were subsequently compared using multiple comparison analyses with Bonferroni correction. Data are presented as means (standard deviation) or median values (range). *P*-values < 0.0001 were considered statistically significant.

ROM, range of motion

**Table S7.** The minimum and maximum values for the gait kinematics of the pelvis angle for the three body height classifications (N = 424)

| **Variables** | **Group 1, 100–120 cm**  **(n = 57)** | **Group 2, 121–140 cm**  **(n = 226)** | **Group 3, ≥ 141 cm**  **(n = 141)** | ***P-value*** | **Effect size (η^2^)** |
| --- | --- | --- | --- | --- | --- |
| Pelvis tilt anterior/posterior stance phase angle (degrees) | Minimum 12.34 (5.06) maximum 14.96 (4.88) | Minimum 13.19 (4.24) maximum 15.93 (4.25) | Minimum 11.89 (4.31) maximum 14.73 (4.20) | Minimum 0.019 maximum 0.025 | Minimum 0.02 maximum 0.02 |
| Pelvis tilt anterior/posterior ROM of gait cycle (degrees) | 3.36 (1.26–5.62) | 3.29 (1.24–9.20) | 3.27 (0.96–6.60) | 0.894 | 0.001 |
| Pelvis obliquity upward/downward stance phase angle (degrees) | Minimum −4.45 (−7.78 to −2.33) maximum 5.22 (3.31–8.60) | Minimum −4.68 (−9.12 to −1.22) maximum 5.53 (1.21–9.51) | Minimum −4.27 (−9.45 to −2.06) maximum 5.18 (2.89–9.46) | Minimum 0.146 maximum 0.262 | Minimum 0.01 maximum 0.01 |
| Pelvis obliquity upward/downward ROM of gait cycle (degrees) | 10.43 (6.35–17.07) | 10.96 (4.00–19.34) | 10.35 (4.65–19.18) | 0.188 | 0.01 |
| Pelvis rotation internal /external rotation angle (degrees) | Minimum −6.69 (−13.80 to −2.65) maximum 7.93 (2.67–14.99) | Minimum −6.55 (−20.10 to −1.13) maximum 7.63 (1.65–19.84) | Minimum −5.93 (−13.57 to −1.17) maximum 6.90 (2.10–16.84) | Minimum 0.050 maximum 0.021 | Minimum 0.01 maximum 0.02 |
| Pelvis rotation internal /external rotation ROM of one gait cycle (degrees) | 14.61 (5.32–28.33) | 14.21 (3.56–39.94) | 12.34 (3.62–30.40) | 0.025 | 0.02 |

Differences between the three groups were analyzed using the Kruskal–Wallis test or one-way analysis of variance depending on the distribution of the data. Data are presented as means (standard deviation) or median values (range). *P*-values < 0.0001 were considered statistically significant.

ROM, range of motion

**Table S8.** The minimum and maximum values for the gait kinematics of the hip angle for the three body height classifications (N = 424)

| **Variables** | **Group 1, 100–120 cm**  **(n = 57)** | **Group 2, 121–140 cm**  **(n = 226)** | **Group 3, ≥ 141 cm**  **(n = 141)** | ***P*-value** | **Effect size (η^2^)** |
| --- | --- | --- | --- | --- | --- |
| Hip flexion/extension stance phase angle (degrees) | Minimum −7.11 (6.42) maximum 38.46 (18.09–53.18) | Minimum −5.34 (4.92) maximum 37.98 (25.41–55.00) | Minimum −6.26 (5.34) maximum 36.37 (22.92–50.67) | Minimum 0.072 maximum  0.006 | Minimum 0.01 maximum 0.02 |
| Hip flexion/extension ROM of gait cycle (degrees) | 47.32 (5.13) | 46.11 (4.93) | 44.25 (5.09) | maximum 1 and 2: < 0.309  1 and 3: < 0.0001  2 and 3: < 0.002 | 0.04 |
| Hip adduction/abduction stance phase angle (degrees) | Minimum −5.61 (2.50) maximum 6.25 (0.08–10.70) | Minimum −6.17 (2.63) maximum 5.80 (−0.83–10.97) | Minimum −6.25 (2.85) maximum 5.43 (−0.42–10.44) | Minimum 0.420 maximum 0.003 | Minimum 0.001 maximum 0.03 |
| Hip adduction/abduction ROM of gait cycle (degrees) | 13.10 (2.61) | 12.87 (2.81) | 12.40 (2.71) | 0.159 | 0.01 |
| Hip internal /external rotation stance phase angle (degrees) | Minimum −10.23 (3.97) maximum 4.75 (4.32) | Minimum −11.73 (4.87) maximum 2.93 (5.03) | Minimum −10.01(4.83) maximum 3.12 (4.72) | Minimum 0.002 maximum 0.039 | Minimum 0.03 maximum 0.02 |
| Hip internal /external rotation ROM of gait cycle (degrees) | 18.92 (4.41) | 20.07 (5.47) | 20.77(5.83) | 0.165 | 0.01 |

Differences between the three groups were analyzed using the Kruskal–Wallis test or one-way analysis of variance depending on the distribution of the data. Variables with significant differences were subsequently compared using multiple comparison analyses with Bonferroni correction. Data are presented as means (standard deviation) or median values (range). *P*-values < 0.0001 were considered statistically significant.

ROM, range of motion

**Table S9.** The minimum and maximum values for the gait kinematics of the knee, ankle, and foot progression angle for the three body height classifications (N = 424)

| **Variables** | **Group 1, 100–120 cm**  **(n = 57)** | **Group 2, 121–140 cm**  **(n = 226)** | **Group 3, ≥ 141 cm**  **(n = 141)** | ***P*-value** | **Effect size (η^2^)** |
| --- | --- | --- | --- | --- | --- |
| Knee flexion/extension stance phase minimum angle (degrees) | 2.12 (4.43) | 3.09 (3.77) | 2.62 (3.70) | 0.183 | 0.01 |
| Knee flexion/extension swing phase maximum angle (degrees) | 60.96 (4.48) | 60.80 (4.66) | 58.33 (4.61) | 1 and 2: < 1.000  1 and 3: < 0.001  B and C: < 0.0001 | 0.06 |
| Knee flexion/extension ROM of gait cycle (degrees) | 60.22 (4.77) | 59.23 (4.74) | 57.31 (5.02) | 1 and 2: < 0.501  1 and 3: < 0.0001  2 and 3: < 0.001 | 0.05 |
| Ankle dorsiflexion/plantarflexion swing phase minimum angle (degrees) | −17.82 (6.68) | −17.44 (5.30) | −17.97 (5.46) | 0.654 | 0.001 |
| Ankle dorsiflexion/plantarflexion stance phase maximum angle (degrees) | 14.79 (3.96) | 14.80 (3.63) | 13.91 (3.39) | 0.060 | 0.01 |
| Ankle dorsiflexion/plantarflexion ROM of gait cycle (degrees) | 32.65 (6.52) | 32.28 (5.26) | 31.97 (5.30) | 0.715 | 0.001 |
| Foot progression internal/external rotation stance phase angle (degrees) | Minimum −6.83 (−20.41−10.19) maximum −0.42 (6.17) | Minimum −6.16 (−26.11−9.11) maximum 0.31 (6.19) | Minimum −5.38 (−21.12−3.55) maximum 0.09 (5.36) | Minimum 0.514 maximum 0.705 | Minimum 0.002 maximum 0.001 |
| Foot progression internal/external rotation ROM of gait cycle (degrees) | 15.56 (8.68–28.11) | 14.39 (5.61–30.15) | 14.66 (6.00–28.81) | 0.022 | 0.02 |

Differences between the three groups were analyzed using the Kruskal–Wallis test or one-way analysis of variance depending on the distribution of the data. Variables with significant differences were subsequently compared using multiple comparison analyses with Bonferroni correction. Data are presented as means (standard deviation) or median values (range). *P*-values < 0.0001 were considered statistically significant.

ROM, range of motion

**Table S10.** The minimum and maximum values for the kinetics of the lower extremity for the three body height classifications (N = 424)

| **Variables** | **Group 1, 100–120 cm**  **(n = 57)** | **Group 2, 121–140 cm**  **(n = 226)** | **Group 3, ≥ 141 cm**  **(n = 141)** | ***P*-value** | **Effect size (η^2^)** |
| --- | --- | --- | --- | --- | --- |
| Sagittal hip minimum moment (Nm/Kg) | −0.60 (−0.94 to −0.21) | −0.63 (−1.10 to −0.32) | −0.63 (−1.11 to −0.31) | 0.301 | 0.01 |
| Sagittal knee maximum moment (Nm/Kg) | 0.42 (0.09–0.79) | 0.51 (0.06–1.43) | 0.50 (0.06–1.02) | 0.019 | 0.02 |
| Sagittal ankle maximum moment (Nm/Kg) | 0.95 (0.76−1.15) | 1.12 (0.58−1.51) | 1.24 (0.60−1.77) | 1 and 2: < 0.0001  1 and 3: < 0.0001  2 and 3: < 0.0001 | 0.32 |
| Sagittal hip maximum power (W/Kg) | 0.72 (0.31–1.70) | 0.82 (0.32–2.36) | 0.84 (0.39–2.09) | 0.104 | 0.01 |
| Sagittal hip power at integral in gait cycle (Nm s/Kg) | 14.87 (8.32–26.47) | 14.97 (6.43–35.58) | 14.97 (8.19–27.50) | 0.848 | 0.001 |
| Sagittal knee minimum power (W/Kg) | −0.51 (−2.14 to −0.13) | −0.56 (−2.35 to −0.01) | −0.56 (−1.74−0.15) | 0.834 | 0.001 |
| Sagittal knee power at integral in gait cycle (Nm s/Kg) | 13.45 (6.28–25.33) | 15.22 (5.04–50.10) | 15.80 (7.78–33.64) | 0.049 | 0.01 |
| Sagittal ankle maximum power (W/Kg) | 2.33 (1.37–4.81) | 2.86 (0.56–5.44) | 2.80 (1.10–4.80) | 0.006 | 0.02 |
| Sagittal ankle power at integral in gait cycle (Nm s/Kg) | 17.92 (12.52–31.38) | 20.99 (6.74–37.54) | 20.67 (9.17–37.36) | 0.006 | 0.02 |

The moment values denote the minimum or maximum moment during the gait cycle. The power values denote the minimum or maximum and integral power over the gait cycle. Units: moment: Nm/kg; power: W/kg. Negative values for hip moments indicate flexion moments. Negative values for power indicate absorption. Differences between the three groups were analyzed using the Kruskal–Wallis test of variance. Variables with significant differences were subsequently compared using multiple comparison analyses with Bonferroni correction. Data are presented as median values (range). *P*-values < 0.0001 were considered statistically significant.

**Supporting Information Captions**

**Table S1.** Temporal parameters, and minimum and maximum values for the gait kinematics of the pelvis and lower extremity for the three age groups.

**Table S2.** The values for the power at integral in the gait cycle for the three age groups.

**Table S3.** Gait Deviation Index for each sex and age group.

**Table S4.** Demographic characteristics of the participants for each body height classification.

**Table S5.** Temporal parameters for each body height classification.

**Table S6.** Spatial parameters and Gait Deviation Index for each body height classification.

**Table S7.** The minimum and maximum values for the gait kinematics of the pelvis angle for the three body height classifications.

**Table S8.** The minimum and maximum values for the gait kinematics of the hip angle for the three body height classifications.

**Table S9.** The minimum and maximum values for the gait kinematics of the knee, ankle, and foot progression angle for the three body height classifications.

**Table S10.** The minimum and maximum values for the kinetics of the lower extremity for the three body height classifications.

**Supplementary data.** Gait cycle data N424 mean value.
